# Continuous Detection of Cerebral Vasodilatation and Vasoconstriction Using Intracranial Pulse Morphological Template Matching

**DOI:** 10.1371/journal.pone.0050795

**Published:** 2012-11-30

**Authors:** Shadnaz Asgari, Nestor Gonzalez, Andrew W. Subudhi, Robert Hamilton, Paul Vespa, Marvin Bergsneider, Robert C. Roach, Xiao Hu

**Affiliations:** 1 Department of Computer Engineering and Computer Science, California State University, Long Beach, California, United States of America; 2 Department of Neurosurgery, University of California Los Angeles, Los Angeles, California, United States of America; 3 Department of Bioengineering, University of California Los Angeles, Los Angeles, California, United States of America; 4 Department of Biology, University of Colorado, Colorado Springs, Colorado, United States of America; 5 Department of Emergency Medicine, University of Colorado Anschutz Medical Campus, Denver, Colorado, United States of America; Kaohsiung Chang Gung Memorial Hospital, Taiwan

## Abstract

Although accurate and continuous assessment of cerebral vasculature status is highly desirable for managing cerebral vascular diseases, no such method exists for current clinical practice. The present work introduces a novel method for real-time detection of cerebral vasodilatation and vasoconstriction using pulse morphological template matching. Templates consisting of morphological metrics of cerebral blood flow velocity (CBFV) pulse, measured at middle cerebral artery using Transcranial Doppler, are obtained by applying a morphological clustering and analysis of intracranial pulse algorithm to the data collected during induced vasodilatation and vasoconstriction in a controlled setting. These templates were then employed to define a vasodilatation index (VDI) and a vasoconstriction index (VCI) for any inquiry data segment as the percentage of the metrics demonstrating a trend consistent with those obtained from the training dataset. The validation of the proposed method on a dataset of CBFV signals of 27 healthy subjects, collected with a similar protocol as that of training dataset, during hyperventilation (and CO_2_ rebreathing tests) shows a sensitivity of 92% (and 82%) for detection of vasodilatation (and vasoconstriction) and the specificity of 90% (and 92%), respectively. Moreover, the proposed method of detection of vasodilatation (vasoconstriction) is capable of rejecting all the cases associated with vasoconstriction (vasodilatation) and outperforms other two conventional techniques by at least 7% for vasodilatation and 19% for vasoconstriction.

## Introduction

Modern strategies for managing patients in neurocritical care units utilize a set of monitoring techniques to evaluate various fluctuating physiological markers to inform intervention decisions on an individual basis [1]. Various monitoring modalities [2], [3] have been introduced in neurocritical units to provide the assessment of cerebral hemodynamics [e.g. cerebral blood flow (CBF) and cerebral blood flow velocity (CBFV)], intracranial hydraulics [e.g. intracranial pressure (ICP)], electrophysiology [e.g. electroencephalography (EEG)], cerebral oxygenation [e.g., partial pressure of oxygen], and brain metabolism [e.g. microdialysis (MD)]. However, the methods currently available to evaluate the pathophysiological changes of the cerebral circulation have significant time resolution limitations and do not allow continuous evaluations of the fluctuations in cerebral perfusion. A modality capable of providing real time information on the changes occurring in the cerebral vasculature could potentially increase the time effectiveness of therapeutic interventions and prevent secondary cerebral damage due to ischemia or hyperperfusion. Such a modality could play a fundamental role in the management of conditions such as cerebral vasospasm after subarachnoid hemorrhage, evaluation of the collateral flow in patients with acute and chronic unstable ischemic stroke and monitoring of cerebrovascular changes associated with traumatic brain injury [4], [5].

Methodologies to assess the cerebral vasculature like Transcranial Doppler (TCD) are limited due to the skull density that only allows insonation of large vessels of the circle of Willis in individuals with favorable windows by trained technicians. While digital subtraction, CT, or MRI angiographic methods provide accurate images of the cerebral vasculature and in some cases functional information of the cerebral blood flow [6], they can only be preformed intermittently and carry risks associated with the use of contrast media, radiation or the endovascular intervention. A few indirect metrics also exist that can be used to assess the cerebral vasculature using hemodynamic concepts such as resistance and vascular tone, e.g. Gosling pulsatility index (PI) [7], Pourcelot resistance index (RI) [8], and critical closing pressure (CCP) and resistance area product (RAP) [9]. Although there is some success of applying them in detecting cerebrovascular changes, these metrics are often not accurate as they are derived from simplified models of cerebral blood flow circulation whose underlying assumptions may not be applicable to the related clinical scenario [10]. In addition to a potential model-mismatch, hemodynamics metrics such as CCP and RAP rely on approximating the cerebral arterial blood pressure using peripherally measured systemic pressures, which may further compromises the accuracy of these metrics due to confounding influence from extracranial systemic circulatory systems.

Our effort has been focused on developing and validating novel methods of analyzing continuously acquired pulsatile signals of an intracranial origin, e.g. ICP or CBFV to derive cerebrovascular metrics that are less influenced by the factors mentioned above. Based on the pulse wave propagation theory [11], we have proposed an intracranial latency model that incorporates pulse transmit time of both intracranial and extracranial pulses so that the confounding influence of extracranial origins on characterizing pulse wave velocity of the cerebral arterial bed can be reduced [12]. We have also shown that the slope of this latency model can successfully track the cerebral vasculature changes relative to those of the systematic arterial bed [13]. However, this latency model cannot detect changes that occur downstream to the intracranial measurement site, e.g., the middle cerebral artery (MCA) if CBFV at the MCA is used, because only the timing of the onset of each pulse is used.

In the present study, we propose a new method for assessing the cerebral vasculature to further address the limitations of existing approaches. This approach is based on the observation that intracranial pressure pulse morphology undergoes a consistent change as patients inhale CO2-enriched gas mixture [14]. This observation leads to two logical premises of this new approach: 1) intracranial pulses including ICP and CBFV originate from vascular pulsations propagating from the heart and hence acute cerebrovascular changes can modulate the shape of these pulses; 2) this modulation induces changes of pulse morphology in an expected fashion, i.e., vasodilatation or vasoconstriction causes pulses to change in a certain way so that a quantification of how well an observation of pulse morphological changes matches this expectation can lead to metrics of cerebral vasodilatation and vasoconstriction.

To quantitatively characterize the pulse shape and its changes, we have developed an algorithm termed **Mo**rphological **C**lustering and **A**nalysis of an **I**ntracranial **P**ulse (MOCAIP) [15]. MOCAIP can robustly deliver an array of metrics (n = 128) to comprehensively characterize the amplitude, curvature, slope, and time-intervals among peaks and troughs of pulses. An observed pulse morphological change can be first quantified in terms of MOCAIP metrics and then compared with a template of expected changes during either cerebral vasodilatation or vasoconstriction to establish the likelihood of conformance of this observation to the template. We name this new approach **P**ulse **M**orphological **T**emplate **M**atching (PMTM) and it can be noted that PMTM is non-parametric, does not depend on any models of CBF circulation, and is not confounded by influences from systemic circulation. In the following, we study and validate the proposed method using data recorded during physiological challenges known to cause cerebral vasodilatation and vasoconstriction.

## Materials and Methods

### Data

The training dataset consists of the CBFV and electrocardiograph (ECG) recordings of five female patients (21, 24, 26, 32 and 54 years old), who were admitted at UCLA Medical Center for the evaluation of headaches potentially due to chronic shunt implantation. During their hospitalization, the patients underwent a CO_2_ challenge test to rule out cerebral vasculature-related headache by inhaling a 5% CO_2_ mixture for less than 3 minutes. Simultaneous cardiovascular monitoring was performed using the bedside GE monitors and CBFV was measured non-invasively at middle cerebral artery (MCA) using TCD machine (Multi-Dop X, Compumedics DWL, Singen, Germany) as part of daily clinical bedside assessment of patients’ cerebral hemodynamics by technicians of with the Cerebral Blood Flow laboratory at UCLA Department of Neurosurgery. Doppler probes were secured to a custom headset to preserve insonation angle and MCA was insonated through the ipsilateral temporal window at depths of the best signal (44–55 mm). CBFV and ECG signals were recorded during the CO_2_ test with few minutes preceding (baseline) and proceeding (post-test) at a sampling rate of 400 Hz using a mobile cart at the bedside that was equipped with the PowerLab TM SP-16 data acquisition system (ADInstruments, Colorado Springs, CO).

Testing dataset were obtained from a portion of a larger study investigating cerebral hemodynamics during exposure to hypobaric hypoxia conducted at the University of Colorado Anschutz Medical Campus, as previously described in [22]. Briefly, 29 healthy individuals (25 males and 4 females; 18 to 44 years) were instrumented to monitor partial pressure of end-tidal carbon dioxide (P_ET_CO2) (Ametek CD-3A, AEI Technologies, Pitsburgh, PA USA), ECG (Bioamp, ADInstruments, Colorado Springs, CO USA), left radial artery blood pressure (Model 7000, Colin Medical Instruments Corp., San Antonio, TX, USA) and MCA flow velocity (Multi-Dop T2, DWL Electronic Systems, Singen, Germany). Using Powerlab instrument (16SP, ADInstruments, Colorado Springs, CO, USA) signals were synchronized and recorded simultaneously at a sampling rate of 1 KHz, but later re-sampled at 400 Hz. After 6 minutes of rest, subjects underwent a series of tests in which fractional concentrations of inspired gases and breathing rate were manipulated to evaluate baseline and normoxic cerebral hemodynamic parameters, including vasoreactivity. Between tests, partial pressure of end-tidal oxygen (P_ET_O_2_) returned to baseline values established at rest. For the present study, we used the data collected during baseline (rest), hyperventilation and CO_2_ rebreathing tests. For hyperventilation, individuals were asked to breathe to a metronome at 18 breaths per minute for 2 minutes in order to reduce P_ET_CO_2_ to approximately 20 mmHg. During the CO_2_ rebreathing test, subjects took 2 to 3 deep breaths of 7.5% CO_2_, 60% O_2_, balance N_2_, then rebreathed through ∼15-L circuit until P_ET_CO2 rose to above 50 mmHg. Complete datasets were recorded in 27 subjects and used for further analysis.

Our entire dataset consisted of data segments from total of 32 subjects where 5 of them were headache patients and 27 of them were healthy subjects.

### Ethics Statement

The five headache patients consented in writing for allowing their data to be analyzed under the protocol as approved by the UCLA Internal Review Board. For healthy subjects, after institutional ethics approval by University of Colorado Anschutz Medical Campus, potential subjects were screened to identify those with no histories of head injuries, migraines, smoking, or medical conditions affected by hypoxia, such as anemia, pregnancy, or hypertension. After obtaining written consent, volunteers were physically examined and excluded if results revealed previously undisclosed medical conditions, or if they were not able to achieve at least 200 W of effort during an incremental cycle ergometer test.

### An Overview of the MOCAIP Algorithm

**Figure 1:**
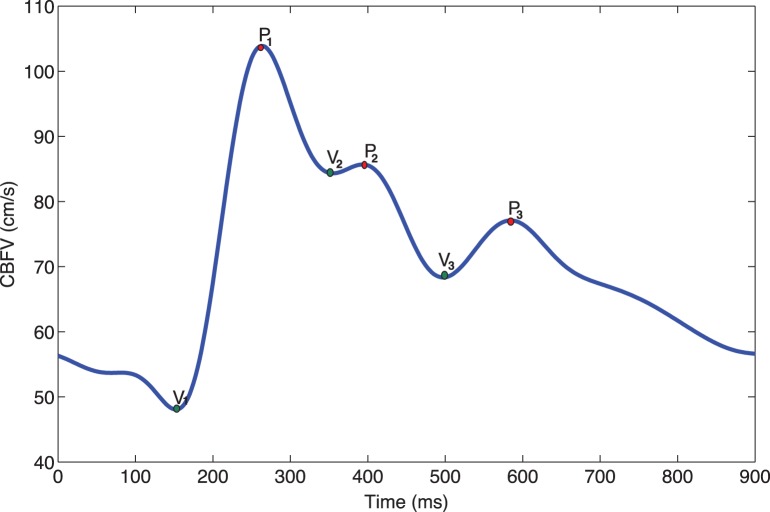
A representative intracranial pulse with its identified peaks and troughs.

MOCAIP is a recently developed paradigm for analyzing pulsatile signals, e.g. ICP and CBFV [15]. This integrated and modular pulse analysis framework uses a pulse extraction technique [11] to segment the continuous signal into a sequence of individual pulses. Then, in order to handle the practical problems of noise and artifacts commonly found in clinical signals, a hierarchical clustering approach [16], different filtration and the correlation calculation of the extracted pulse with all the pulses of a reference library of already validated pulses, are employed. A set of the peak candidates or curve inflections are detected for each validated pulse and then the three sub-peaks are identified from these peak candidates by maximizing the probability of observing the current sub-peaks given the prior Gaussian distributions of the peaks (learned from the library of valid pulses). Finally, as table 1 summarizes, 128 pulse morphological metrics are extracted using the identified peaks and troughs of the pulse (a representative intracranial pulse is shown in figure 1).

**Table 1:** The description of 128 metrics derived from the six landmarks detected by MOCAIP algorithm on a pulse of CBFV (The zero of time axis refers to the timing of R component of electrocardiograph QRS).

Metric group index	Notation	Description
1	dV_1_, dV_2_, dV_3_, dP_1_, dP_2_, dP_3_	Amplitude of landmark relative to the minimum point prior to initial rise
2	L_V1P1_, L_V1P2_, L_V1P3_, L_V2P2_, L_V3P3_	Time delay among landmarks
3	Curv_v1_, Curv_v2_, Curv_v3_, Curv_p1_, Curv_p2_, Curv_p3_	Absolute curvature of each landmark
4	K_1_, K_2_, K_3_, RC_1_, RC_2_, RC_3_	K_1_, K_2_, K_3_ are slope of each rising edge and RC_1_, RC_2_, RC_3_ are time-constants of each descending edge
5	mCBFV, dias CBFV	Mean CBFV and diastolic CBFV
6	L_T_	Time delay of V_1_ to ECG QRS peak
7	mCurv	Mean absolute curvature of the pulse
8	WaveAmp	Maximum among dP_1_ and dP_3_
9	dP_p1p2,_ ….	Ratio among landmark amplitudes
10	L_V1P1_/L_T_,…	Ratio among time delays
11	Curv_v1_/Curv_v2_,…	Ratio among curvatures
12	*K*_1_/*RC*_1,_…	Ratio among slopes/RCs

The 28 metrics belonging to group indices of 1 to 8 are called basic metrics, while the remaining 100 metrics (belonging to group indices of 9 to 12 ) are extended metrics calculated as ratios among basic metrics within each group.

Further improvement of each of MOCAIP individual processing blocks has been the focus of some of our recent studies, e.g. enhancement of the performance of valid pulse recognition applying a singular value decomposition based algorithm [17], [18] or increase of the accuracy of peak designation using a nonlinear regression-based method [19], an integrated peak recognition technique [20], and a non-parametric Bayesian tracking algorithm [21]. Therefore, the current version of MOCAIP algorithm can be reliably applied to process continuous signal recordings from real clinical environment to extract useful morphological features of the corresponding pulses.

### Pulse Morphological Template Matching (PMTM)

#### Template construction

The core of the PMTM approach is to obtain the template that defines the expected pulse morphological change pattern associated with cerebral vasodilatation and vasoconstriction. In the present work, we use a training data set to identify set of MOCAIP metrics that consistently increase/decrease during vasodilatation but decrease/increase during vasoconstriction as such a template. The qualification of this template can be established by the fact that vasodilatation and vasoconstriction are opposite physiological processes and hence the direction of change of a valid indicator of vasodilatation or vasoconstriction should be opposite in these two conditions.

Let us assume that an experiment is first conducted to induce paired cerebral vasodilatation-vasoconstriction in multiple subjects. Intracranial pulses from episodes of vasodilatation and vasoconstriction are then analyzed by MOCAIP. A monotonic trending detection algorithm is used next to determine the direction of changes for each MOCAIP metric during vasodilatation and vasoconstriction, which are coded as 1 or −1 representing “increase” and “decrease”, respectively. To proceed, we define consistent vasodilatation metrics as those that undergo changes in the same direction across all subjects enrolled in the experiment during vasodilatation. We define vector 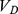 where
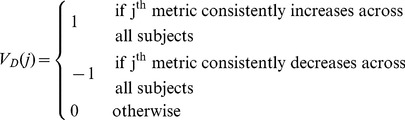
(1)to represent for each MOCAIP metric its consistency and the direction of change. A similar vector 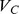 can be defined for vasoconstriction. With this definition, we can then identify the PMTM template as the subset (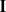) of indices of the MOCAIP metrics whose corresponding elements in 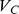 and 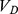 have opposite signs, i.e., 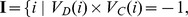 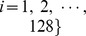.

In summary, 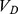, 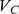, and 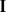 are the three elements of a PMTM template. In the next subsection, we describe how to apply a PMTM template to detect either cerebral vasodilatation or vasoconstriction.

#### Application of the template

Let us assume we are given a segment of an intracranial pulsatile signal and asked whether this signal is associated with vasodilatation, vasoconstriction, or neither of them. We define a vasodilatation index (VDI) and a vasoconstriction index (VCI) as approximate likelihood of vasodilatation and vasoconstriction, respectively. VDI and VCI can be calculated following the steps below:

- Apply MOCAIP to analyze this pulsatile intracranial signal and obtain a time series of MOCAIP metrics.
- Use the same monotonic trending detection algorithm as adopted in the training phase to determine the existence and the direction of the change of each MOCAIP metric. The result can be expressed as a vector 
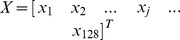
 where
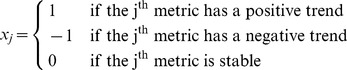
(2)Note: a new outcome for trend detection is introduced that is “stable”.
- Define 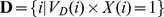 and 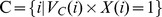.
- Then VDI can be calculated as 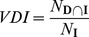, and 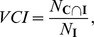 where 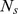 denotes the number of elements within a set 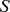 and 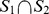 denotes the intersect of set 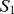 and 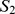

#### Monotonic trend detection

In the present work, we adopted a simple trend detection algorithm. In particular, we used a robust line fitting algorithm [14] to conduct a weighted least square fit of each MOCAIP metric time series with a line. In constructing the PMTM template, we simply take the sign of the resultant slope to indicate whether the trend is positive or negative because the consistency or existence of the trend for 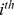
metric will be verified by criterion 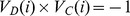. In applying the template to a new signal segment, we need to first determine whether a trend exists. This is done by using the *p* value of the estimated slope, i.e., a trend exists if the corresponding *p value* is less than a pre-set threshold. We will discuss an approach to determine this threshold later on in the paper. Once establishing whether a trend exists, the sign of the slope is taken to judge the direction of the change as needed in equation (2). This algorithm was also adopted in our recent work [14] and has been shown to be effective in robustly discovering monotonic trend in MOCAIP metrics.

### Data Analysis and Validation Protocol

#### Differentiating between cerebral vasodilatation/vasoconstriction and baseline

The data segments corresponding to the rising edge of CBFV during the CO_2_ challenge test of 5 headache patients were treated as vasodilating episodes while data segments corresponding to the falling edge of CBFV during post-test measurements were analyzed as vasoconstricting episodes. These edges can be readily marked because the change of mean CBFV was significant from the baseline to the maximal value during CO_2_ challenge and back from the maximal value to the baseline once patients started to breathe from the room air.

According to the procedure to build PMTM template as described before, a pre-set threshold is needed for establishing whether a MOCAIP metric trend exists. This is handled by picking the threshold to correspond to the 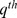 percentile of all *p* values from line-fitting of all training segments. To explore the effect of *q* on the performance of the proposed method, we changed it as 10^th^, 30^th^, 50^th^, 70^th^ and 90^th^ percentiles. At each *q* value, we sweep a threshold from 1 to 0 at a step size of 0.01 to obtain a binary outcome from VDI/VCI values. This results in conventional Receiver Operator Characteristics (ROC) curves for VDI and VCI, respectively. The following parameters were then calculated from a ROC curve: 1) the area under the ROC curve (*AUC*), its standard deviation *(SD)* and 95% confidence interval (*CI*); 2) the partial area under the ROC curve for True Positive Rate (TPR) greater than 0.8 (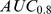); 3) the value of False Positive Rate (FPR) when TPR = 0.80 (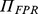). Moreover, the operational point is determined from a ROC curve as the point on the curve closest to the point [0, 1]. The threshold value at this point will be used to provide a binary outcome for a given VDI/VCI value.

#### Correlation analysis of VDI and VCI with cerebrovascular resistance index

It is known that during a cerebral vasomotor reactivity test such as CO_2_ rebreathing (hyperventilation), the dilatation (constriction) of cerebral vessels would result in a decrease (increase) of cerebrovascular resistance. To further evaluate the efficacy of the proposed indices (VDI and VCI) as a measure of vasoreactivity, we also studied the correlation of the calculated indices and cerebrovascular resistance index (CVRi); a conventional measure of cerebrovascular resistance. For this purpose, arterial blood pressure (ABP) pulses of the testing dataset were delineated similar to those of CBFV and then the ratios of beat-by-beat mean ABP and mean CBFV were obtained. 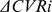 is computed as the change in the value of CVRi at the beginning and end of the test 
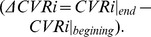
 Finally, the vector of calculated VDI (VCI) for all the subjects are correlated with that of 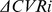 during CO_2_ rebreathing (hyperventilation) and the Pearson correlation coefficients are obtained.

#### Determining the ground truth for detection of vasodilatation and vasoconstriction

Since establishing the ground truth of the vasodilatation and vasoconstriction cases via direct observation of arterial caliber changes is almost impossible, for the present work, the ground truth is determined based on the expected trend of change in P_ET_CO2 and CVRi during vasodilatation and vasoconstriction. For this purpose, 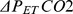 is computed as the change in the value of observed P_ET_CO2 at the beginning and end of the test 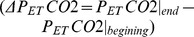. In addition, 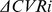 is calculated as described in previous subsection. Then a data segment during CO_2_ rebreathing is considered as a true vasodilatation case unless both P_ET_CO2 and CVRi for that data segment do not follow the expected trend of increasing and decreasing, respectively (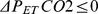 and 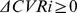 ). Similarly, a data segment during hyperventilation is considered as a true vasoconstriction case unless 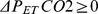 and 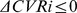.

#### Comparison with critical closing pressure and resistance area product

We compare the performance of the proposed method of detection of vasoreactivity using VDI/VCI with that of two other conventional hemodynamic metrics, i.e. resistance area product (RAP) and critical closing pressure (CCP). For this purpose, beat-by-beat RAP and CCP values are calculated by applying first harmonic fitting technique [23] to ABP and CBFV pulses of testing dataset. Then, 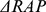 and 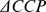
are computed by subtracting the RAP and CCP values at the beginning of the data segment from that at the end of the data segment. Finally, the ROC curves of vasoreactivity are obtained by applying 100 equally spaced threshold points (swept from the minimum to maximum values of 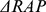 and 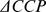) to compute binary outcomes for detection of vasodilatation and vasoconstriction. These ROC curves are compared with those of the proposed method.

#### The effect of the selection of training dataset

We compare the performance between using the template constructed from the headache patients and that from the normal subjects. This is done by fixing the number of training subjects to be 5 and then randomly sample 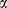 sets of 5-subject training data from all qualified normal subjects, i.e., those with both validated episodes of vasodilatation and vasoconstriction. The performance of using the template from normal subjects will be the average of the performance metrics from these 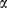 sets.

We also test the influence of varying the number of subjects in the training dataset on the size of the obtained template (number of consistent MOCAIP metrics in the template) and accuracy of vasoreactivity detection. For this purpose, we merge the data from headache patients and normal subjects and then use an increasing number of training subjects to build the template and test it on the remaining subjects. Again, we randomly pick at most 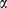 sets of *n*-subject training data where *n* ranges from 1 to total number of qualified subjects. If possible number of permutations is less than 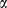, all permutations are used.

## Results

The duration of the collected CBFV data for the headache patients was (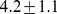
minute). This data included (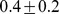
minute) of resting baseline, (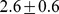
minute) of CO_2_ challenge test and (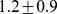
minute) of post-test measurements. The percentage of change in the mean CBFV value from resting to CO_2_ challenge test was 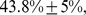 indicating a significant cerebral vasodilatation during the challenge test. The collected data for the healthy subjects included (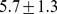
min) of resting baseline, (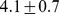
min) of CO_2_ rebreathing and (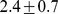
min) of hyperventilation.

**Figure 2:**
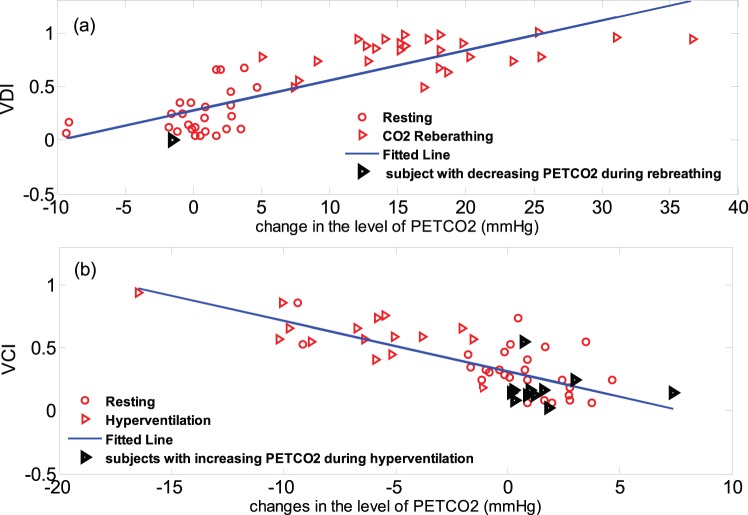
Correlation analysis of the proposed vasoreactivity indices and the level of change of P_ET_CO2 (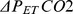); (a) calculated vasodilatation index (VDI) over 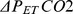 for all 27 subjects during baseline and CO_2_ rebreathing measurement; (b) calculated vasoconstriction index (VCI) over 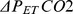 for all 27 subjects during baseline and hyperventilation measurement.

The plot of the calculated VDI for testing data segments of CO_2_ rebreathing and baseline measurements over their corresponding level of change in P_ET_CO2 (figure 2-a) demonstrates that an increase in the level of P_ET_CO2 is accompanied by an increase in the calculated VDI. (Correlation coefficient of 0.82 and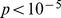). Similarly, as figure 2-b demonstrates, a decrease in the value of P_ET_CO2 during hyperventilation results in an increase in the value of calculated VCI (correlation coefficient of −0.75 and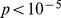).

**Figure 3:**
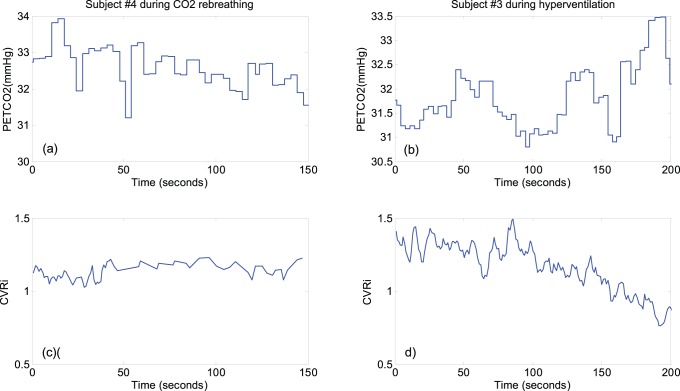
Examples of invalid data segments during CO_2_ rebreathing and hyperventilation; (a) non-increasing trend of P_ET_CO2 for subject #4 during CO_2_ rebreathing; (b) non-decreasing trend of P_ET_CO2 for subject #3 during hyperventilation; (c) non-decreasing trend of cerebrovascular resistance index (CVRi) for subject #4 during CO_2_ rebreathing; (d) non-increasing trend of CVRi for subject #3 during hyperventilation.

**Figure 4:**
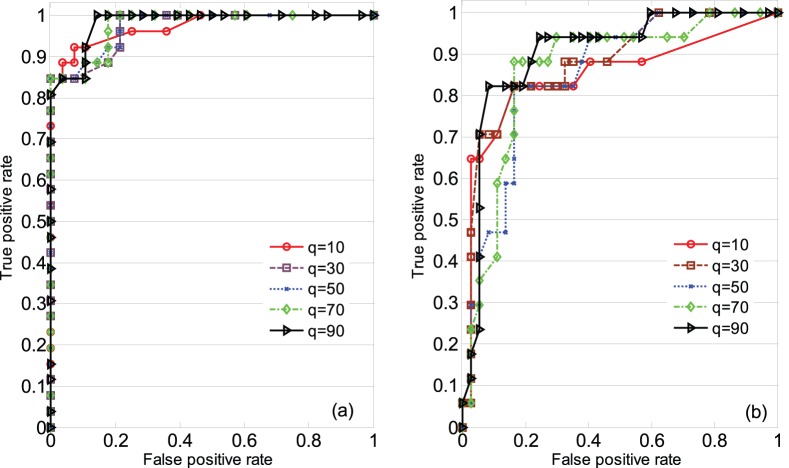
Detection of vasodilatation and vasoconstriction applying the proposed method on the testing dataset; (a) ROC curves for detection of vasodilatation; (b) ROC curves for detection of vasoconstriction. ROC curves are obtained using 10^th^, 30^th^, 50^th^, 70th and 90^th^ percentile of the probabilities of t-statistics (calculated from the regression lines fitted to the data points of the corresponding metric) over the subjects in the training dataset. Each point on the ROC curve is resulted from systematically changing the threshold 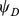 from 1 to 0 with decremental steps of 0.01.

Following the procedure described before, the ground truth for detection of vasodilatation and vasoconstriction were determined by studying the trend of change of P_ET_CO2 and cerebrovascular resistance index during CO_2_ rebreathing and hyperventilation episodes. This investigation revealed that during CO_2_ rebreathing test P_ET_CO2 increases (for at least 5 mmHg) for all the subjects in the study (figure 2-a), except for subject #4 whose data point is highlighted with bolder triangle on that figure. Moreover, the calculated CVRi for this subject does not follow the expected decreasing trend during CO_2_ rebreathing (figure 3-c). As CO_2_ is a potent cerebral vasodilator, we conclude that the data segment of this subject during CO_2_ rebreathing is not a valid representation of a vasodilatation event. Similarly, we observe that 11 subjects had an unexpected increase in the level of P_ET_CO2 during hyperventilation (highlighted with bolder triangle points on figure 2-b). We believe that these subjects had difficulty with long periods of hyperventilation. They started the hyperventilation with a correct breathing rate, but after a while, they got fatigued and lightheaded. So they began to breathe more slowly to accommodate and as a result, their P_ET_CO2 drifted back up. Investigating the trend of CVRi during hyperventilation reveals that in fact for 10 of these subjects, the calculated cerebrovascular resistance index also did not demonstrate an increasing trend expected during a vasoconstriction event (figure 4-b and 4-d show the P_ET_CO2 and CVRi for one of the 10 subjects). Therefore, the data segments of these 10 subjects during hyperventilation could not be a valid representative of a vasoconstriction episode. In summary, based on our determined ground truth, only 16 healthy subjects had both validated data segments of vasoconstriction and vasodilatation.

The ROC curves for detection of vasodilatation employing the determined ground truth are plotted in figure 4-a and the related calculated parameters are presented in table 2. We observe that although the estimated area under the ROC curves for different values of 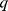 (as explained earlier in the paper) are all above 0.97 and have small standard deviations, the proposed method seems to perform the best when 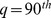 percentile. The calculation of the operational point for the ROC curve of 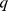 = 90 results in a FPR of 0.10 and TPR of 0.92 at detecting cerebral vasodilatation using VDI >0.53. Similarly, the ROC curves and their related parameters for detection of vasoconstriction are demonstrated in figure 4-b and table 3, respectively. For the ROC curve of 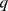 = 90, the area under the curve is 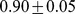 and the calculation of the operational point results in a FPR = 0.08 and TPR = 0.82 at detecting cerebral vasoconstriction using VCI >0.53.

**Table 2:** The calculated parameters for the ROC curves of detection of vasodilatation (figure 2-b) applying the proposed method on the testing dataset.

*q*	10	30	50	70	90
*AUC* *	0.97(0.02)	0.97(0.02)	0.97(0.02)	0.97(0.02)	0.98(0.01)
*CI* **	(0.92,1)	(0.92,1)	(0.92,1)	(0.93,1)	(0.94,1)
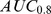 x	0.97	0.97	0.97	0.97	0.98
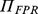 xx	0	0	0	0	0

* Area under the ROC curve.

** 95% confidence interval.

x Partial area under the ROC curve for 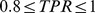.

xx Value of FPR when 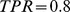.

**Table 3:** The calculated parameters for the ROC curves of detection of vasoconstriction (figure 3-b) applying the proposed method on the testing dataset.

*q*	10	30	50	70	90
*AUC* *	0.84 (0.06)	0.88(0.05)	0.85(0.06)	0.86(0.06)	0.90(0.05)
*CI* **	(0.72,0.97)	(0.76,0.99)	(0.72,0.97)	(0.74,0.98)	(0.79,1)
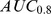 x	0.75	0.78	0.78	0.79	0.87
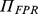 xx	0.16	0.16	0.16	0.16	0.08

* Area under the ROC curve.

** 95% confidence interval.

x Partial area under the ROC curve for 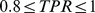.

xx Value of FPR when 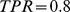.

**Figure 5:**
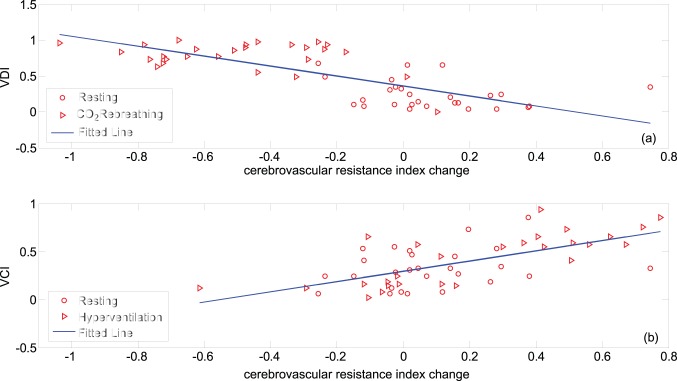
Correlation analysis of the proposed vasoreactivity indices and cerebrovascular resistance index change (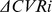); (a) calculated vasodilatation index (VDI) over 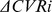 during CO2 rebreathing; (b) calculated vasoconstriction index (VCI) over 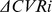 during hyperventilation.

The plot of the calculated VDI (VCI) for testing data segments of CO_2_ rebreathing (hyperventilation) and baseline measurements over their corresponding level of change in CVRi (figure 5) shows that a higher change in the CVRi during CO_2_ rebreathing (hyperventilation) would correspond to higher values of VDI (VCI). The correlation coefficient between VDI (VCI) and 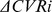 during CO_2_ rebreathing (hyperventilation) is −0.74 (0.62) with 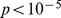.

**Figure 6:**
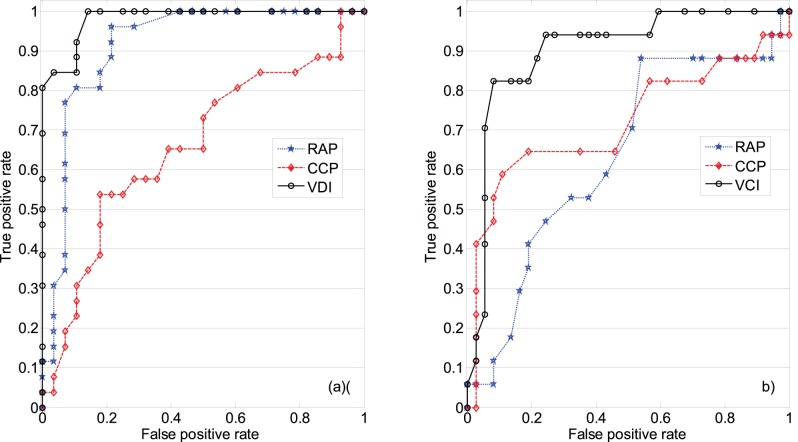
Comparison of vasoreactivity detection performance using the proposed vasoreactivity indices and two other conventional hemodynamic metrics; resistance area product (RAP) and critical closing pressure (CCP); (a) vasodilatation; (b) vasoconstriction.

Figure 6 illustrates the result of detection of vasodilatation and vasoconstriction employing the proposed vasoreactivity indices with 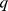 = 90 and two other conventional hemodynamic metrics (RAP and CCP). We observe that the ROC curve of the proposed method is above the other two. In fact, the areas under the ROC curves for detection of vasodilatation using the proposed method, RAP and CCP are 0.98, 0.91 and 0.65, respectively. For vasoconstriction, these areas are 0.90, 0.63 and 0.71, respectively. Therefore, the proposed method outperforms the other two by at least 7% for vasodilatation and 19% for vasoconstriction.

The investigation of the effect of the selection of the training dataset on the detection of cerebral vasodilatation with 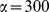 resulted in 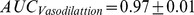 and 
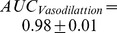
, for the training dataset of 5 headache subjects (template size of 49 metrics) and 5 healthy subjects (average template size of 19 metrics), respectively. The same analysis for detection of vasoconstriction resulted in 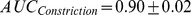 and 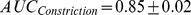 for the training dataset of 5 headache subjects versus that of 5 healthy subjects. We observe that the selection of the healthy subjects for the training dataset may slightly improve the detection of vasodilatation, but it worsens the detection of vasoconstriction.

**Figure 7:**
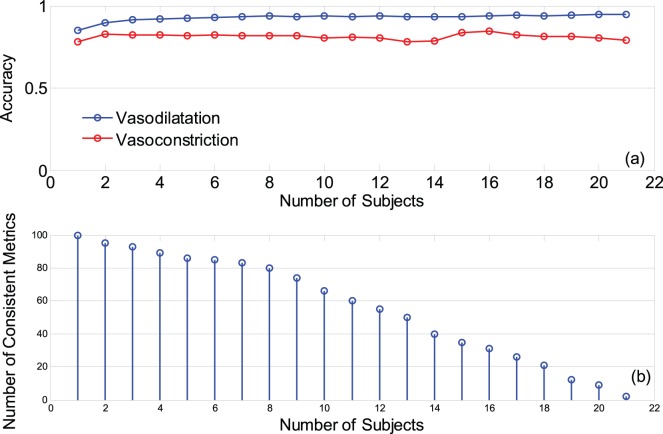
The effect of training dataset on the detection of vasoreactivity; (a) accuracy of the detection of vasodilatation and vasoconstriction for different number of subjects in the training dataset; (b) The size (number of consistent MOCAIP metrics) of the largest template obtained from a training dataset of n-subject where n = 1,…,21.

Figure 7-a illustrates the effect of the size of the training dataset on the performance of the proposed method. As the number of subjects in the training dataset increases the accuracy of the detection of vasodilatation increases from 0.84 (when 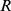 = 1) to 0.92 (for 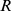 = 6), but then further enlargement of the training dataset does not affect the performance of detection. Similarly the initial enlargement of the training dataset (up to 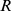 = 6) improves the accuracy of detection of vasoconstriction, and then the performance slightly degrades (with a rate less than 1% per added subject) till 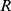 = 13. For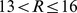, the performance enhances (by rate of 2% per added subject) and again starts to decrease with slow rate of 1% per added subject.

Figure 7-b shows the size of the largest template obtained from a training dataset of n-subject (n = 1,…,21). We observe that when all 21 subjects are included in the training dataset, our PMTM template would consist of only 2 MOCAIP metrics (mean ICP and diastolic pressure point). But by excluding one subject from the training dataset (in this case normal subject # 15), the PMTM template size increases to 9 at its largest (for n = 20) and includes 6 metrics with an increasing/decreasing trend during vasodilatation/vasoconstriction (dP_2_, dV_2_, mICP, diasP, K_1_/RC_1_, RC_3_/RC_1_) and 3 metrics with decreasing/increasing trend during vasodilatation/vasoconstriction (dV_1_/dV_2_, dP_1_/dV_2_ and RC_1_/RC_2_). Further exclusion of another subject (normal subject #27) from the training dataset results in a larger PMTM template of 12 metrics (for n = 19) which includes three latency ratio metrics (L_V1P1_/L_V1P3_, L_V1P1_/L_P1P3_ and L_V1P3_/L_P1P3_), in addition to the aforementioned 9 metrics. We conclude that these 12 metrics are good indicators of vasoreactivity. In fact, detection of vasodilatation and vasoconstriction employing the proposed method with this template (of 12 metrics) and the comparison threshold of (VDI or VCI>0.57) results in accuracy of 94% and 88%, respectively. Please note that excluding more number of subjects from the training dataset would produce a larger template, but aiming at obtaining a template made from a large training dataset with good accuracy for detection of vasoreactivity, the aforementioned template of 12 metrics seems to be a reasonable solution.

## Discussion

In the present work, two CBFV-based novel indices (VDI and VCI) have been proposed to for real-time detection of cerebral arterial vasodilatation and vasoconstriction, respectively. These two indices in essence quantify the matching degree between a template, which is a set of common CBFV pulse morphological metrics that consistently increase/decrease during a vasodilatation or vasoconstriction process for a set of training subjects, and the set of morphological metrics that increase/decrease for a testing CBFV segment. The results (figures 4-a and 4-b) showed that these two indices can detect vasodilatation and vasoconstriction with an accuracy of 0.92 and 0.82, respectively.

### Intracranial Pulse Morphology and the Cerebral Vascular Changes

The proposed PMTM method fundamentally departures from existing approaches of characterizing cerebrovascular changes in terms of avoiding both simplified assumptions of the cerebral hemodynamics that are needed in model based approaches [7] and approximating cerebral arterial blood pressure using extracranial systemic blood pressure [24], [25].Instead, the PMTM heavily depends on characterizing morphological changes of intracranial pulse waveforms to detect cerebrovascular changes. Intracranial pulses such as ICP and CBFV originate from the transmitted systemic pulses and are under the influence of the cerebrovascular changes. Acute changes in the diameter, tone, and compliance of the cerebrovascular are reflected in the changes of the pulse shape. However, an analytic description of how the shape changes are related to cerebrovascular changes does not exist. Hence, the PMTM uses a data-driven approach to establish a template that characterizes the expected changes of pulse morphology in terms of 128 metrics derived from MOCAIP analysis. In this way, the accuracy of detecting cerebrovascular changes will only depend on the quality of the training data that implicitly define the changing patterns of pulse morphology in response to cerebrovascular changes, which are acute cerebral vasodilatation and vasoconstriction as studied in the present work.

Indeed, some metrics of pulse morphology of systemic arterial blood pressure pulses have been already used to characterize properties of the systemic arterial bed such as compliance and resistance [26], [27]. However, a single metric may not be sufficient to reflect complex morphological patterns associated with acute cerebral vascular changes. There are indications in our previous studies that intracranial pulse morphological metrics are able to reflect cerebral vascular and hemodynamic changes. In a study of using ICP pulse waveforms to classify cerebral perfusion status [28], it was found that elevation of the third peak of an ICP pulse may be associated with low global CBF. In a recent study [14], we have reported the existence of consistent changes of ICP pulse waveforms induced by hypercapnic cerebral vasodilatation.

Further evidence of the potential of using pulse waveform changes to inference cerebral vascular changes has been obtained in our investigation of the association between ICP pulse waveform changes and cerebral Lactate Pyruvate ratio (LPR) increase [29]. The present work is built upon these existing studies by proposing an algorithm to conduct the detection of vasodilatation and vasoconstriction using only intracranial pulse morphological features.

### Performance of PMTM

Performance of the PMTM was rigorously studied using two independent data sets. Using the template built from five patients with severe headache undergoing CO_2_ challenge test, the performance of detecting vasodilatation for 27 normal subjects achieved a TPR of 0.92 and a FPR of 0.10. Detecting vasoconstriction has an inferior TPR of 0.82. It is inherently challenging to establish the ground truth of the vasodilatation and vasoconstriction cases because a direct observation of arterial caliber changes is almost impossible. In the present work, we primarily rely on the well-known physiological influence of CO2 on dilating the cerebral arteries to identify true vasodilatation cases. However, it is more ambiguous to identify true vasoconstriction cases from the hyperventilation dataset. We therefore use a conventional metric CVRi as a cross-reference to filter out some obviously false vasoconstriction cases. This practice may result in a large amount of uncertain vasoconstriction cases and eventually affect the performance of VCI.

On the other hand, it is important to point out that the proposed approach will never misclassify a vasodilatation case for a vasoconstriction case and vice versa. This can be guaranteed by using a threshold greater than 0.5 on VDI and VCI because the summation of VDI and VCI is strictly less or equal to one. This means that an inquiry data segment can only be judged to be either a vasodilatation case or a vasoconstriction case. Indeed, ROC curve analysis shows that the operating threshold for both VDI and VCI is slightly greater than 0.5.

### Training Data Set

Employing the training dataset of headache subjects (versus healthy subjects) resulted in a higher performance of detection of vasoconstriction, while it did not significantly affect that of the vasodilatation. While the template obtained from the training dataset of five headache subjects included 49 MOCAIP metrics, the average number of such metrics (over 300 selected copies of trainings) for 5 healthy subjects was 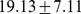. This result indicates that that the changes of CBFV pulse morphology in response to vasodilatation/vasoconstriction in the headache subjects were more consistent than those of the healthy subjects. Moreover, it is possible that even some of the 16 normal subjects with the expected decreasing trend in the level of P_ET_CO2 were unable to maintain the target rate and/or depth of breathing during the hyperventilation phase to cause a significant vasoconstriction (e.g. subject# 15 and 27 from figure 7-b). So training dataset of healthy subjects (specially the vasoconstriction segments) may not result in an accurate representation of the pulse morphology templates and this can cause a degraded performance for detection of vasoconstriction.

Enlargement of the training dataset may have a two-fold effect on the performance of the proposed method of detection; adding more subjects to the training dataset may increase the accuracy of detection of vasodilatation/vasoconstriction by diversifying the training set, but it may also impair the performance by limiting the number of metrics in the PMTM template (As the training dataset become larger, the number of consistent metrics over all subjects decreases). Our results show that size of the training dataset does not affect the good performance of the detection of vasodilatation as long as it includes the data of at least 5 subjects. Moreover, although the performance of detection of vasoconstriction may slightly change depending on the size of the training dataset, the level of change is small (less than 1% decrease in accuracy per added subject).

Finally, the PMTM template of 12 metrics (dP_2_, dV_2_, mICP, diasP, K_1_/RC_1_, RC_3_/RC_1,_ L_V1P1_/L_V1P3_, L_V1P1_/L_P1P3_, L_V1P3_/L_P1P3_, dV_1_/dV_2_, dP_1_/dV_2_ and RC_1_/RC_2_) was obtained from a training dataset of 19 subjects and demonstrated a high accuracy in detection of vasoreactivity. Therefore, this template is a recommended template to be prospectively evaluated for future studies.

### Potential Limitations of the Approach

Simple monotonic trend detection is currently used to build the PMTM template. This apparently cannot handle more complex changes of MOCAIP metrics in response to cerebral vasodilatation and vasoconstriction. Furthermore, the amount and the rate of change of MOCAIP metrics are not considered in the template building process. However, it can be also argued that adopting a simple trending pattern is a key reason for success in determining a set of consistent metrics to form the template because such a simple pattern is more likely to be shared by different subjects given the complex relationship between CO2 and CBFV exists in individual subjects [30].

To realize the full potential of achieving a continuous detection of acute cerebral vascular changes by the bedside, further studies are needed to test the proposed method and determine its efficacy in TBI and SAH patients undergoing neurocritical care. To be able to continuously monitor cerebral vasoreactivity, ICP pulse is more appropriate and this would require the construction of an ICP template following the same approach descried here for CBFV pulses. In addition, a new set of training data may be required to build the template when applying the proposed method to different patient populations.

## References

[1] Wartenberg KE, Schmidt JM, Mayer SA (2007) Multimodality monitoring in neurocritical care. Crit Care Clin 23: 507–538. 17900483 10.1016/j.ccc.2007.06.002

[2] Tisdall MM, Smith M (2007) Multimodal monitoring in traumatic brain injury: current status and future directions. Br J Anaesth 99: 61–67. 17548431 10.1093/bja/aem143

[3] Diedler J, Czosnyka M (2010) Merits and pitfalls of multimodality brain monitoring. Neurocrit Care 12: 313–316. 20390380 10.1007/s12028-010-9350-5

[4] Klingelhofer J, Sander D, Hakk K, Schwarze J, Dressnandt J, et al (1996) Relationships between delayed ischemic dysfunctions and intracranial hemodynamics following subarachnoid hemorrhage. J Neurol Sci 143: 72–78. 8981301 10.1016/s0022-510x(96)00138-4

[5] Mayberg MR, Batjer HH, Dacey R, Diringer M, Haley EC, et al (1994) Guidelines for the management of aneurysmal subarachnoid hemorrhage. A statement for healthcare professionals from a special writing group of the Stroke Council, American Heart Association. Circulation 90: 2592–2605. 7955232 10.1161/01.cir.90.5.2592

[6] Satoh T, Onoda K, Tsuchimoto S (2003) Visualization of intraaneurysmal flow patterns with transluminal flow images of 3D MR angiograms in conjunction with aneurysmal configurations. AJNR Am J Neuroradiol 24: 1436–1445. 12917142 PMC7973683

[7] Beasley MG, Blau JN, Gosling RG (1979) Changes in internal carotid artery flow velocities with cerebral vasodilation and constriction. Stroke 10: 331–335. 462522 10.1161/01.str.10.3.331

[8] Arbeille P, Roncin A, Berson M, Patat F, Pourcelot L (1987) Exploration of the fetal cerebral blood flow by duplex Doppler–linear array system in normal and pathological pregnancies. Ultrasound Med Biol 13: 329–337. 3303590 10.1016/0301-5629(87)90166-9

[9] Bloor BM (1972) Cerebral hemodynamics. The critical closing pressure. Bull Soc Int Chir 31: 311–317. 4627492

[10] Panerai RB (2003) The critical closing pressure of the cerebral circulation. Med Eng Phys 25: 621–632. 12900178 10.1016/s1350-4533(03)00027-4

[11] Hu X, Xu P, Lee DJ, Vespa P, Baldwin K, et al (2008) An algorithm for extracting intracranial pressure latency relative to electrocardiogram R wave. Physiol Meas 29: 459–471. 18354246 10.1088/0967-3334/29/4/004 PMC2629794

[12] Hu X, Subudhi AW, Xu P, Asgari S, Roach RC, et al (2009) Inferring cerebrovascular changes from latencies of systemic and intracranial pulses: a model-based latency subtraction algorithm. J Cereb Blood Flow Metab 29: 688–697. 19142194 10.1038/jcbfm.2008.160 PMC2664398

[13] Asgari S, Subudhi AW, Roach RC, Liebeskind DS, Bergsneider M, et al (2011) An extended model of intracranial latency facilitates non-invasive detection of cerebrovascular changes. J Neurosci Methods 197: 171–179. 21310179 10.1016/j.jneumeth.2011.01.032 PMC3072962

[14] Asgari S, Bergsneider M, Hamilton R, Vespa P, Hu X (2011) Consistent changes in intracranial pressure waveform morphology induced by acute hypercapnic cerebral vasodilatation. Neurocrit Care 15: 55–62. 21052864 10.1007/s12028-010-9463-x PMC3130848

[15] Hu X, Xu P, Scalzo F, Vespa P, Bergsneider M (2009) Morphological clustering and analysis of continuous intracranial pressure. IEEE Trans Biomed Eng 56: 696–705. 19272879 10.1109/TBME.2008.2008636 PMC2673331

[16] Kaufman L, Rousseeuw PJ (2005) Finding groups in data : an introduction to cluster analysis: Wiley-Interscience.

[17] Asgari S, Xu P, Bergsneider M, Hu X (2009) A subspace decomposition approach toward recognizing valid pulsatile signals. Physiol Meas 30: 1211–1225. 19794232 10.1088/0967-3334/30/11/006 PMC2851740

[18] Asgari S, Bergsneider M, Hu X (2010) A robust approach toward recognizing valid arterial-blood-pressure pulses. IEEE Trans Inf Technol Biomed 14: 166–172. 19884099 10.1109/TITB.2009.2034845 PMC2887660

[19] Scalzo F, Xu P, Asgari S, Bergsneider M, Hu X (2009) Regression analysis for peak designation in pulsatile pressure signals. Medical & Biological Engineering & Computing 47: 967–977. 19578916 10.1007/s11517-009-0505-5 PMC2734262

[20] Scalzo F, Asgari S, Kim S, Bergsneider M, Hu X (2010) Robust peak recognition in intracranial pressure signals. Biomed Eng Online 9: 61. 20959014 10.1186/1475-925X-9-61 PMC2984490

[21] Scalzo F, Asgari S, Kim S, Bergsneider M, Hu X (2011) Bayesian Tracking of Intracranial Pressure Signal Morphology. Artificial Intelligence in Medicine: Epub ahead of print. 10.1016/j.artmed.2011.08.007 PMC3288115 21968205

[22] Subudhi AW, Panerai RB, Roach RC (2010) Effects of hypobaric hypoxia on cerebral autoregulation. Stroke 41: 641–646. 20185774 10.1161/STROKEAHA.109.574749

[23] Panerai RB, Salinet AS, Brodie FG, Robinson TG (2011) The influence of calculation method on estimates of cerebral critical closing pressure. Physiol Meas 32: 467–482. 21403183 10.1088/0967-3334/32/4/007

[24] Carey BJ, Eames PJ, Panerai RB, Potter JF (2001) Carbon dioxide, critical closing pressure and cerebral haemodynamics prior to vasovagal syncope in humans. Clin Sci (Lond) 101: 351–358. 11566072

[25] Evans DH, Levene MI, Shortland DB, Archer LN (1988) Resistance index, blood flow velocity, and resistance-area product in the cerebral arteries of very low birth weight infants during the first week of life. Ultrasound Med Biol 14: 103–110. 3279689 10.1016/0301-5629(88)90176-7

[26] Wilkinson IB, Fuchs SA, Jansen IM, Spratt JC, Murray GD, et al (1998) Reproducibility of pulse wave velocity and augmentation index measured by pulse wave analysis. J Hypertens 16: 2079–2084. 9886900 10.1097/00004872-199816121-00033

[27] Thiele RH, Durieux ME (2011) Arterial waveform analysis for the anesthesiologist: past, present, and future concepts. Anesth Analg 113: 766–776. 21890890 10.1213/ANE.0b013e31822773ec

[28] Hu X, Glenn T, Scalzo F, Bergsneider M, Sarkiss C, et al (2010) Intracranial pressure pulse morphological features improved detection of decreased cerebral blood flow. Physiol Meas 31: 679–695. 20348611 10.1088/0967-3334/31/5/006 PMC2855777

[29] Asgari S, Vespa P, Bergsneider M, Hu X (2011) Lack of consistent intracranial pressure pulse morphological changes during episodes of microdialysis lactate/pyruvate ratio increase. Physiol Meas 32: 1639–1651. 21904021 10.1088/0967-3334/32/10/011 PMC3334323

[30] Battisti-Charbonney A, Fisher J, Duffin J (2011) The cerebrovascular response to carbon dioxide in humans. J Physiol 589: 3039–3048. 21521758 10.1113/jphysiol.2011.206052 PMC3139085

